# Lobohedleolide suppresses hepatitis C virus replication via JNK/c-Jun-C/EBP-mediated down-regulation of cyclooxygenase-2 expression

**DOI:** 10.1038/s41598-018-26999-w

**Published:** 2018-06-06

**Authors:** Chun-Kuang Lin, Chin-Kai Tseng, Chih-Chuang Liaw, Chiung-Yao Huang, Chih-Ku Wei, Jyh-Horng Sheu, Jin-Ching Lee

**Affiliations:** 1 Doctoral Degree Program in Marine Biotechnology, College of Marine Sciences, National Sun Yat-Sen University, Kaohsiung, Taiwan; 2 Doctoral Degree Program in Marine Biotechnology, Academia Sinica, Taipei, Taiwan; 3 Institute of Basic Medical Sciences, College of Medicine, National Cheng Kung University, Tainan, Taiwan; 4 Center of Infectious Disease and Signaling Research, College of Medicine, National Cheng Kung University, Tainan, Taiwan; 5 Department of Marine Biotechnology and Resources, College of Marine Sciences, National Sun Yat-Sen University, Kaohsiung, Taiwan; 6 Department of Biotechnology, College of Life Science, Kaohsiung Medical University, Kaohsiung, Taiwan; 7 Department of Medical Research, China Medical University Hospital, China Medical University, Taichung, Taiwan; 8 Graduate Institute of Medicine, College of Medicine, Kaohsiung Medical University, Kaohsiung, Taiwan; 9 Research Center for Natural Products and Drug Development, Kaohsiung Medical University, Kaohsiung, Taiwan; 10 Department of Medical Research, Kaohsiung Medical University Hospital, Kaohsiung, Taiwan

## Abstract

Hepatitis C virus (HCV) chronically infects 2–3% people of the global population, which leads to liver cirrhosis and hepatocellular carcinoma. Drug resistance remains a serious problem that limits the effectiveness of US Food and Drug Administration (FDA)-approved direct-acting antiviral (DAA) drugs against HCV proteins. The objective of our study was to discover new antivirals from natural products to supplement current therapeutics. We demonstrated that lobohedleolide, isolated from the Formosan soft coral *Lobophytum crassum*, significantly reduced HCV replication in replicon cells and JFH-1 infection system, with EC_50_ values of 10 ± 0.56 and 22 ± 0.75 μM, respectively, at non-toxic concentrations. We further observed that the inhibitory effect of lobohedleolide on HCV replication is due to suppression of HCV-induced cyclooxygenase-2 (COX-2) expression. Based on deletion-mutant analysis of the COX-2 promoter, we identified CCAAT/enhancer-binding protein (C/EBP) as a key transcription factor for the down-regulation of COX-2 by lobohedleolide, through which lobohedleolide decreased the phosphorylation of c-Jun NH2-terminal protein kinase and c-Jun to suppress HCV-induced C/EBP expression. The combination treatment of lobohedleolide with clinically used HCV drugs synergistically reduced HCV RNA replication, indicating that lobohedleolide exhibited a high biomedical potential to be used as a supplementary therapeutic agent to control HCV infection.

## Introduction

Hepatitis C virus (HCV) is a pathogen of high risk causing chronic liver diseases, including hepatic fibrosis, liver cirrhosis and hepatocellular carcinoma (HCC)^1^. The current clinical standard therapy for chronic hepatitis C (CHC) infection is a combination treatment with pegylated interferon-α (peg-IFN-α) and ribavirin (RBV), but the sustained virologic response (SVR) with only about 40% for HCV genotype 1 patients. As of 2011, two US Food and Drug Administration (FDA)-approved direct-acting antiviral (DAA) agents, boceprevir and telaprevir, against the viral protease activity were shown to significantly improve the antiviral response rate in the treatment of CHC infection^2^. However, the lack of pangenotypic activity and the rapid selection of drug-resistant viral mutants have limited the antiviral response in patients infected with HCV genotypes 2–6^3^. More recently, the second-generation drugs, such as simeprevir and sofosbuvir, were shown to exhibit an improved resistance profile and resulted in a higher cure rate and fewer side effects in patients treated for CHC infection^4,5^. However, due to the high cost of these drugs, some countries might face a potential difficulty in accepting the therapy^6^. Therefore, there still remains a need to discover new anti-HCV agents to improve the current therapy.

HCV is an enveloped RNA virus and belongs to the *Flaviviridae* family^7^. The RNA encodes a single polyprotein consisting of approximately 3,000 amino acids which is cleaved into 10 viral proteins by viral and host proteases^8^. In HCV-related liver diseases, cyclooxygenase-2 (COX-2) and its metabolite prostaglandin E_2_ (PGE_2_), are highly expressed and considered as two of the major cellular effectors causing tissue injury, fibrogenesis, chronic hepatitis, liver cirrhosis, and HCC^9,10^. Several reports and our previous studies have demonstrated that inactivation of COX-2 by COX-2 shRNA or a specific inhibitor can impair HCV replication^11,12^. Therefore, the interruption of HCV-induced COX-2 signaling pathway has been considered as a promising strategy to decrease HCV infection and HCV-induced inflammatory pathogenesis^12,13^.

COX-2 is a rate-limiting enzyme that converts arachidonic acid into prostanoids^14^. The expression of COX-2 is tightly controlled in most of the tissues and is induced at the sites of inflammation by several extracellular and intracellular stimuli, including reactive oxygen species, chemicals, and viral infections. Both nuclear factor-κB (NF-κB) and mitogen-activated protein kinase (MAPK) signaling pathways have been well characterized to activate COX-2 transcription^15^. The central transcriptional region of COX-2 contains several binding sites of transcriptional factors for its expression, including NF-κB, CCAAT/enhancer-binding protein (C/EBP), and activator factor-1 (AP-1)/cyclic adenosine monophosphate (cAMP)-response element (CRE)^16,17^.

Soft corals of the genus *Lobophytum* have been demonstrated to consist of a rich harvest of cembranoids and steroids, which exhibit anti-HIV and anti-inflammatory bioactive propertise^18,19^. Previous studies have shown that lobohedleolide, isolated from the Formosan soft coral *Lobophytum crassum*, exhibited anti-inflammatory activity through the suppression of COX-2 expression *in vitro and in vivo*^19,20^. Therefore, we investigated the effect of lobohedleolide on HCV replication and further clarified whether the molecular mechanisms were down-regulation of HCV-induced COX-2 expression.

## Results

### Lobohedleolide decreases HCV replication in both HCV replicon cells and the infection system

**Figure 1:**
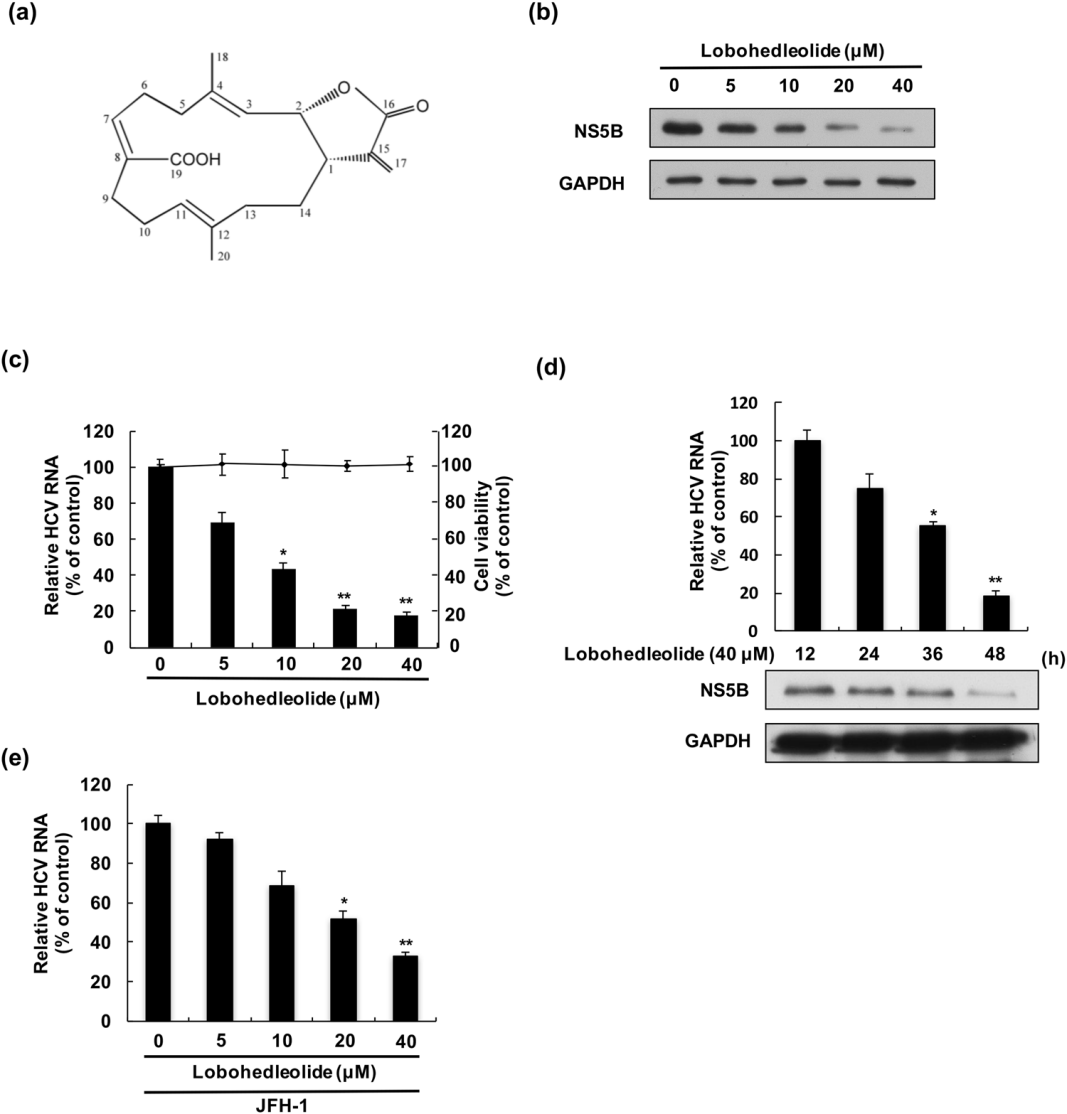
The inhibitory effect of lobohedleolide on HCV replication. (**a**) The chemical structure of lobohedleolide. The reduction effect of lobohedleolide on HCV (**b**) protein synthesis and (**c**) RNA replication. Ava5 cells were treated with lobohedleolide at the indicated concentrations for 3 days. Cell viability was evaluated by MTS assay in the lobohedleolide-treated Ava5 cells. (**d**) Time-dependent inhibition of HCV protein synthesis and RNA replication by lobohedleolide. Ava5 cells were treated with 40 μM of lobohedleolide for the indicated time points. Total cell lysates were subjected to Western blotting with anti-HCV NS5B and anti-GAPDH antibodies. GAPDH served as an equal loading control in Western blotting. (**e**) Concentration-dependent inhibition of lobohedleolide in HCV JFH-1 infection system. Huh7.5 cells were infected with HCV JFH-1 at an MOI of 0.1 for 4 h, and the HCV-infected cells were treated with lobohedleolide at the indicated concentrations for another 3 days. Total cellular RNA was extracted, and the levels of HCV RNA were determined by qRT-PCR following the normalization of cellular *gapdh* mRNA level. The efficiency of inhibition was calculated as the percentage of control (0.1% DMSO treatment). Data are presented as mean ± SD of at least three independent experiments, with each measurement carried out in triplicate. Asterisks indicate significant difference in each sample of lobohedleolide-treated cells at the indicated concentration singly in comparison with DMSO-treated cells. *P < 0.05; **P < 0.01.

To investigate the effect of LH on HCV protein synthesis and RNA replication, HCV replicon cells, Ava5, were incubated with lobohedleolide at different concentrations (5–40 μM) for 3 days. The total cell lysate and cellular RNA were extracted and subjected to Western blotting and qRT-PCR, respectively. The results indicated that lobohedleolide dose-dependently reduced HCV protein and RNA levels, with an EC_50_ value of 10 ± 0.56 μM, compared with those of lobohedleolide -untreated cells (Fig. 1b,c). To rule out the possibility that the inhibitory effect of lobohedleolide on viral replication was due to the cytotoxic effect on the treated cells, the cell viability of lobohedleolide -treated cells was evaluated by MTS assay. As shown in Fig. 1c, no significant cytotoxicity was observed in the lobohedleolide-treated Ava5 cells at effective antiviral concentrations. Furthermore, lobohedleolide time-dependently reduced HCV protein synthesis and RNA replication (Fig. 1d). The anti-HCV activity of lobohedleolide was further evaluated in the HCV JFH-1 infection system, which showed an EC_50_ value of 22 ± 0.75 μM (Fig. 1e).

### Lobohedleolide suppresses HCV replication by inhibiting HCV-induced COX-2 expression and its activity

**Figure 2:**
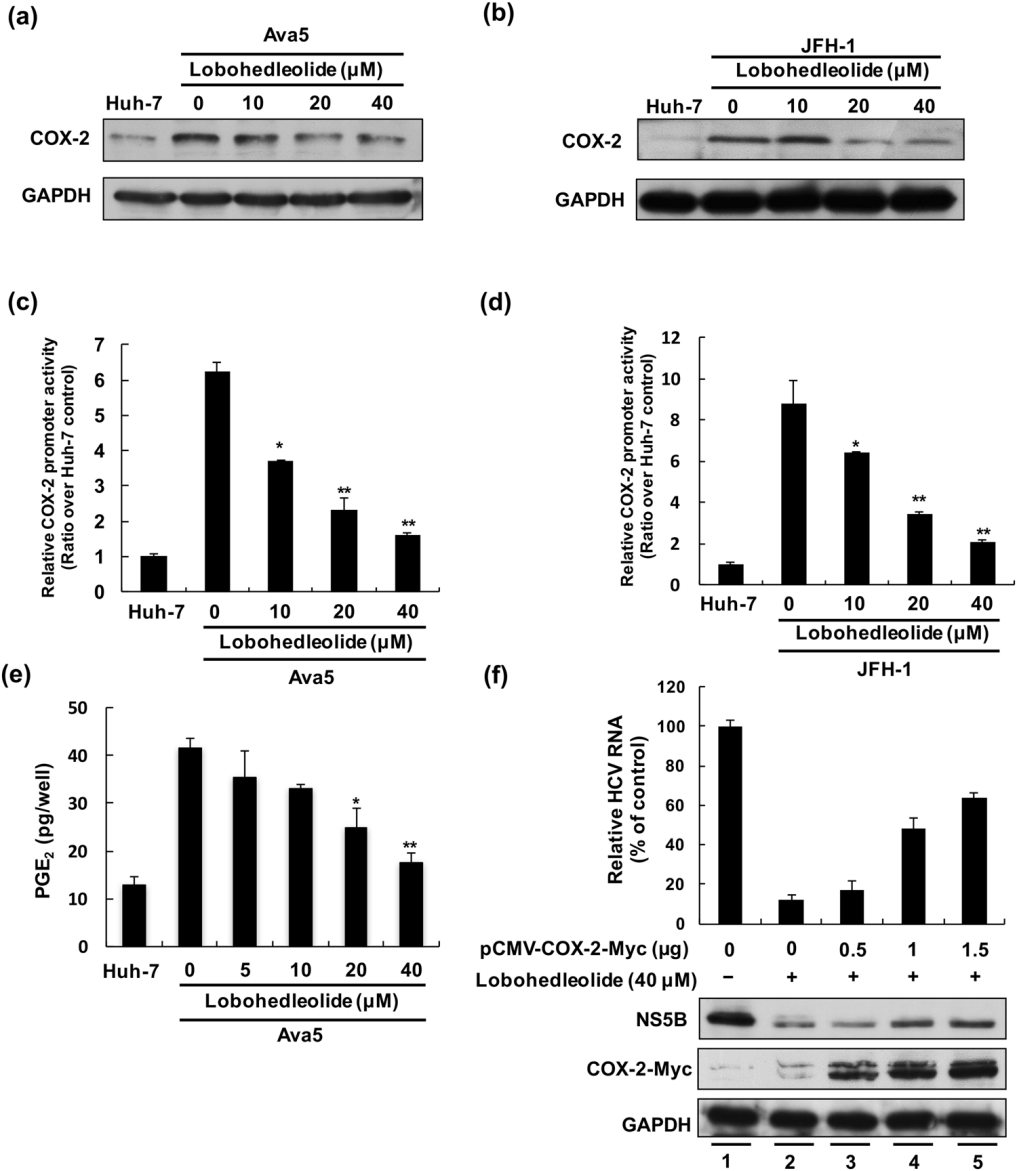
The inhibitory effect of lobohedleolide on HCV-induced COX-2 expression at the protein and transcription levels. The inhibition of HCV-induced COX-2 expression by lobohedleolide in (**a**) HCV replicon cells and (**b**) HCV infection assay. Ava5 cells and HCV JFH-1-infected Huh7.5 cells were treated with lobohedleolide at the indicated concentrations for 3 days. Cell lysates were subjected to Western blotting with antibodies against COX-2 and GAPDH. GAPDH served as an equal loading control in Western blotting. The reduction of HCV-induced COX-2 promoter activity by lobohedleolide in (**c**) HCV replicon cells and (**d**) HCV infection system. Huh-7 cells and Ava5 cells were transfected with pCOX-2-Luc reporter plasmid encoding the firefly luciferase gene under the COX-2 promoter control. The pCOX-2-Luc-transfected Huh-7.5 cells were infected with JFH-1 virus at an MOI of 0.1 for 4 h. The transfected Ava5 cells and JFH-1-infected Huh-7.5 cells were treated with lobohedleolide at the indicated concentrations for 3 days. Cell lysates were subjected to luciferase activity assay. (**e**) Dose-dependent reduction of HCV-induced PGE_2_ production by lobohedleolide. Ava5 cells were incubated with lobohedleolide at the indicated concentrations for 3 days. PGE_2_ production was analyzed by PGE_2_ ELISA kit. (**f**) Concentration-dependent restoration of HCV replication by exogenous COX-2 expression in lobohedleolide-treated Ava5 cells. Ava5 cells were transfected with the indicated amount of pCMV-COX-2-Myc encoding *cox-2* (lanes 3, 4, and 5) or pcDNA3.1 (1.5 μg, lanes 1 and 2) for 6 h, and the transfected cells were treated with DMSO (lane 1) or lobohedleolide at 40 μM for 3 days (lanes 2, 3, 4, and 5). Cell lysates were subjected to Western blotting with antibodies against NS5B, Myc, and GAPDH. GAPDH served as an equal loading control in Western blotting. Total cellular RNAs were extracted and subjected to qRT-PCR to evaluate the level of HCV RNA. Data are presented as mean ± SD of at least three independent experiments, with each measurement carried out in triplicate. Asterisks indicate significant difference in each sample of lobohedleolide-treated cells at the indicated concentration singly in comparison with DMSO-treated cells. *P < 0.05; **P < 0.01.

Inhibition of COX-2 expression has been reported to interfere with HCV replication^21^. In the LPS-induced inflammatory model, lobohedleolide has been shown to exert an inhibitory effect on COX-2 expression^20^. To investigate whether the suppression of COX-2 plays an important role in the activity of lobohedleolide against HCV replication, we first detected the amount of COX-2 protein in the HCV self-replicating cells, Ava5, and HCV JFH-1-infected Huh-7 cells after lobohedleolide treatment. The results of Western blotting indicated that lobohedleolide markedly suppressed the HCV-induced COX-2 protein levels in a dose-dependent manner compared with that in the parental Huh-7 cells, lobohedleolide-untreated Ava5 cells, and HCV-infected Huh-7 cells in the absence of lobohedleolide treatment (Fig. 2a,b). Next, we used a COX-2 promoter-based reporter assay to evaluate the inhibitory effect of lobohedleolide on COX-2 at the transcriptional level. The pCOX-2-Luc, a plasmid encoding firefly luciferase under the control of the COX-2 promoter, was transfected into Ava5 cells or HCV-infected Huh-7 cells, and then, these plasmid-transfected cells were incubated with lobohedleolide at increasing concentrations for 3 days. As shown in Fig. 2c,d, lobohedleolide dose-dependently decreased the HCV-elevated COX-2 promoter activity in Ava5 and HCV-infected cells. We further investigated the effect of lobohedleolide on COX-2 catalytic activity by monitoring the levels of PGE_2_ in Huh-7 and Ava5 cells. The levels of HCV-elevated PGE_2_ were significantly reduced with lobohedleolide treatment in a dose-dependent manner compared with that in the untreated cells (Fig. 2e).

To characterize whether the lobohedleolide -mediated down-regulation of COX-2 expression and its catalytic activity is involved in the suppression of HCV replication, we overexpressed exogenous COX-2 to evaluate the inhibitory activity of lobohedleolide on HCV replication. Ava5 cells were transfected with vehicle or various concentrations of pCMV-COX-2-Myc vector encoding the *cox-2* gene, and the plasmid-transfected cells were incubated with 40 μM of lobohedleolide or 0.1% of DMSO, as a negative control. As shown in Fig. 2f, the lobohedleolide-reduced viral RNA level (top panel, lane 2) was gradually rescued following the increasing expression of exogenous COX-2-Myc (top panel, lanes 3–5) compared with that in the vehicle-transfected Ava5 cells incubated with DMSO (top panel, lane 1). Consistently, the exogenous expression of COX-2 attenuated the inhibitory effect of lobohedleolide on the HCV protein levels, as analyzed by Western blotting. These results clearly indicated that lobohedleolide inhibited HCV replication by down-regulating the virus-induced COX-2 expression.

### Lobohedleolide inhibits C/EBP transcription factor activity

**Figure 3:**
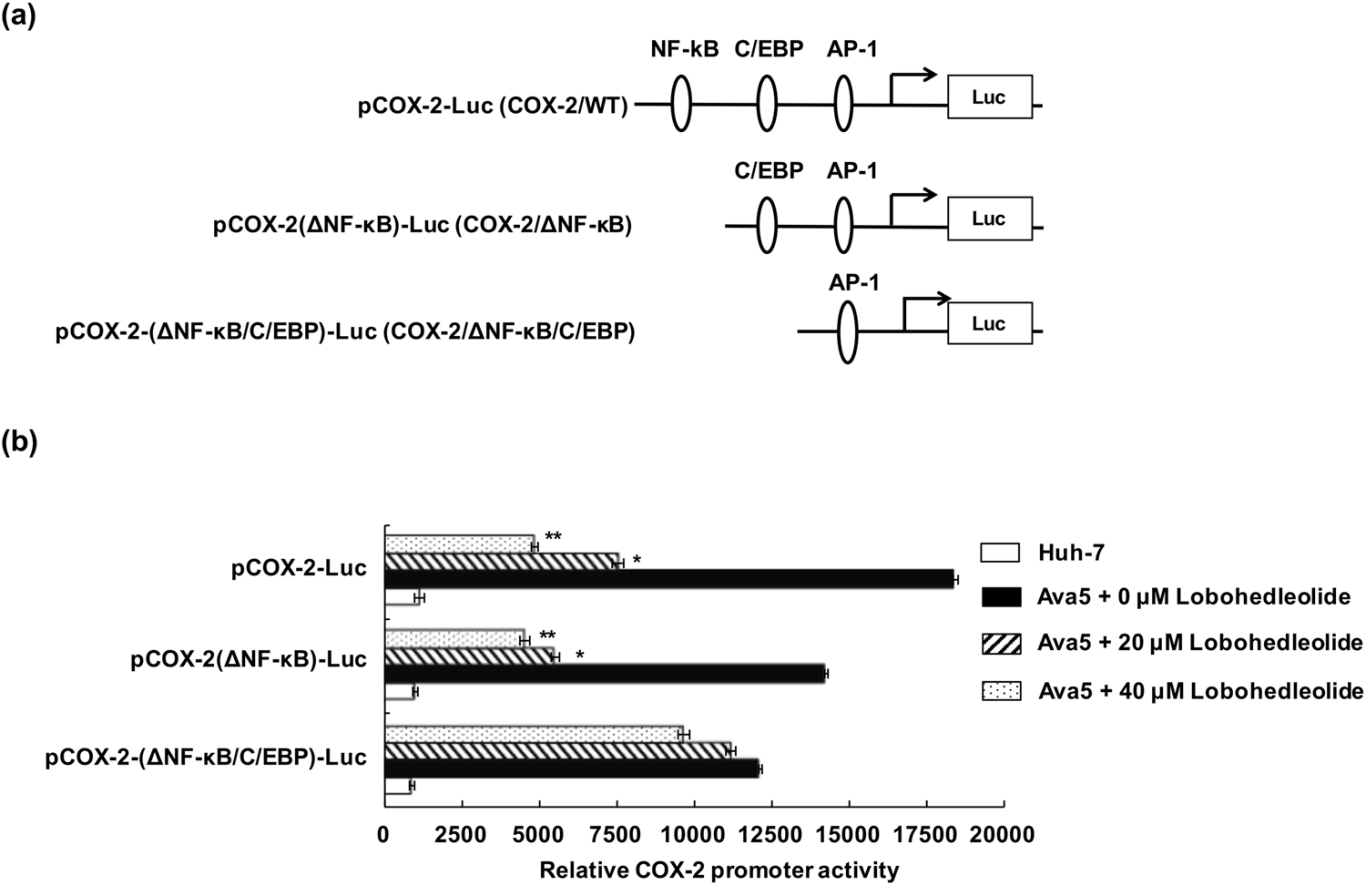
The effect of lobohedleolide on the transcriptional factor activity on COX-2 promoter. (**a**) Schematic diagrams of the deletion of COX-2 promoter constructs. (**b**) Effect of lobohedleolide on the deletion constructs of COX-2 promoter. Ava5 cells were cotransfected with 0.2 μg of pCMV-Rellina-Luc and pCOX-2-Luc, pCOX-2(ΔNF-κB)-Luc, or pCOX-2(ΔNF-κB/C/EBP)-Luc for 6 h. The transfected cells were treated with DMSO or lobohedleolide at 20 and 40 μM for 3 days. Cell lysates were subjected to luciferase activity assay. Data are presented as mean ± SD of at least three independent experiments, with each measurement carried out in triplicate. Asterisks indicate significant difference between lobohedleolide- and DMSO-treated Ava5 cells. *P < 0.05; **P < 0.01.

**Figure 4:**
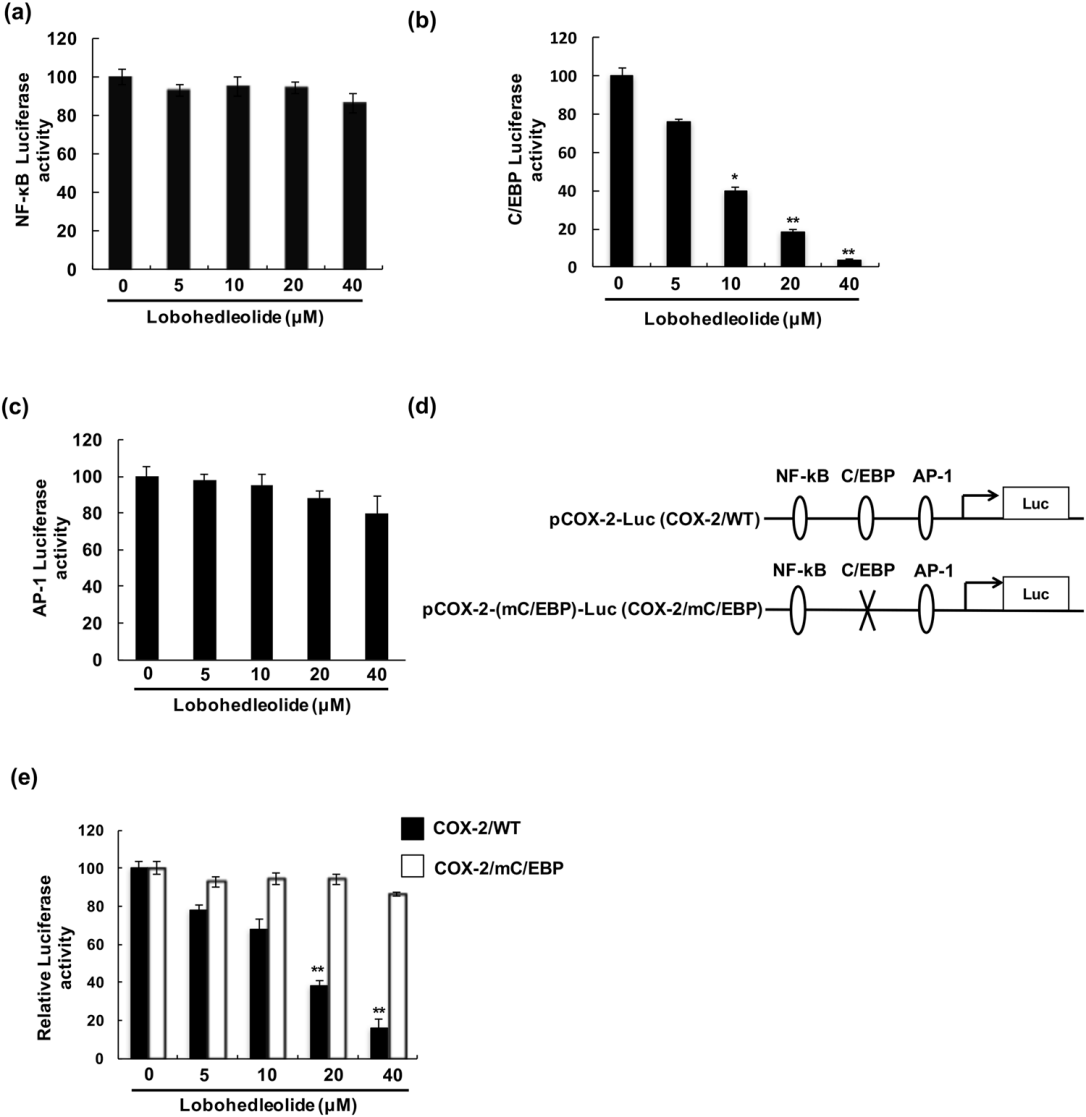
The reduction effect of lobohedleolide on C/EBP transcription factor activity. Ava5 cells were cotransfected with 0.2 μg of pCMV-Rellina-Luc and (**a**) pNF-κB-Luc, (**b**) pC/EBP-Luc, or (**c**) pAP-1-Luc constructs for 6 h, and the transfected cells were treated with lobohedleolide at the indicated concentrations for 3 days. Cell lysates were subjected to luciferase activity assay. (**d**) Schematic diagram of the COX-2 and mutagenized C/EBP of COX-2 promoter constructs. (**e**) The inhibitory effect of lobohedleolide on COX-2 promoter activity was reduced by mutagenized C/EBP on COX-2 promoter. Ava5 cells were transfected with pCOX-2-Luc or pCOX-2-(m/C/EBP)-Luc for 6 h, and the transfected cells were treated with lobohedleolide at indicated concentrations for 3 days. Cell lysates were subjected to luciferase activity assay. Data are presented as mean ± SD of at least three independent experiments, with each measurement carried out in triplicate. Asterisks indicate significant difference between lobohedleolide- and DMSO-treated Ava5 cells. *P < 0.05; **P < 0.01.

COX-2 induction is mediated by multiple transcription factor pathways, such as NF-κB, C/EBP, and AP-1^22^. To identify which binding elements of transcription factors on the COX-2 promoter are responsible for modulating the lobohedleolide -mediated inhibition of COX-2 expression, we created a series of COX-2 promoter reporter constructs carrying the deleted promoter fragments, including wild-type (WT), ΔNF-κB, and ΔNF-κB/C/EBP, linked to firefly luciferase gene to elucidate the effect of lobohedleolide on COX-2 transcription, in which the WT promoter region contained NF-κB, C/EBP, and AP-1 responsive elements (Fig. 3a). Huh-7 and Ava5 cells were transiently transfected with pCOX-2-Luc, pCOX-2(ΔNF-κB)-Luc, or pCOX-2(ΔNF-κB/C/EBP)-Luc, and the plasmid-transfected cells were respectively treated with lobohedleolide at 0, 20 and 40 μM. The relative COX-2 promoter activities were analyzed using luciferase activity assay. As shown in Fig. 3b, lobohedleolide dose-dependently reduced the HCV-induced luciferase activity driven by the COX-2/WT promoter and the COX-2/ΔNF-κB promoter, but exhibited no significant effect on that of luciferase activity driven by the COX-2/ΔNF-κB/C/EBP promoter, suggesting that the transcriptional element C/EBP was critical in the suppression of COX-2 promoter activity by lobohedleolide. To further verify which transcription factors are directly involved in the reduction effect of lobohedleolide on COX-2 promoter activity, Ava5 cells were transfected with individual luciferase reporter vector carrying each transcription element, including NF-κB, C/EBP, and AP-1. The results of the reporter assay showed that lobohedleolide significantly reduced the HCV-induced luciferase activity mediated by C/EBP (Fig. 4b), but exhibited no significant effect on that of luciferase activity driven by NF-κB or AP-1 (Fig. 4a,c). To confirm the inhibitory effect of lobohedleolide on the transcription factor activity of C/EBP in Ava5 cells, we created a COX-2 promoter reporter construct with a point mutation on C/EBP site alone, named as pCOX-2-(mC/EBP)-Luc (Fig. 4d). Ava5 cells were transfected with pCOX-2-Luc or pCOX-2-(mC/EBP)-Luc, and then the plasmid-transfected cells were incubated with lobohedleolide at concentrations ranging from 5 to 40 μM. As shown in Fig. 4e, the COX-2/mC/EBP promoter activity was not affected by lobohedleolide compared with COX-2/WT promoter activity, confirming that the C/EBP transcription factor contributed to the suppression of HCV-induced COX-2 expression by lobohedleolide.

### Lobohedleolide reduces HCV-induced C/EBPβ binding and expression, c-Jun phosphorylation, and the activation of JNK

**Figure 5:**
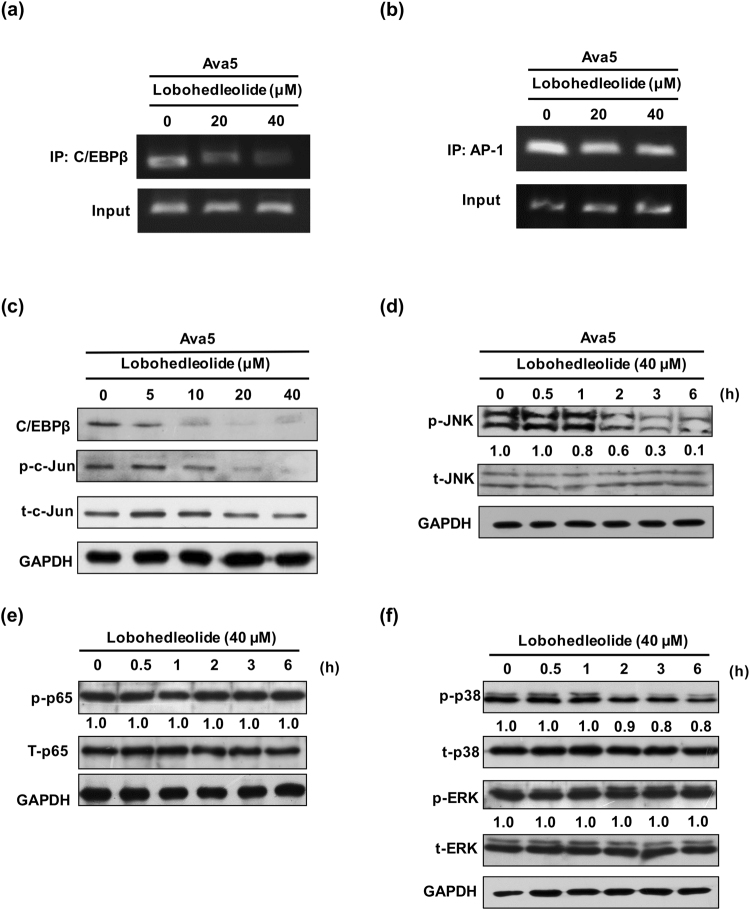
The reduction effect of lobohedleolide on C/EBPβ binding and expression, c-Jun phosphorylation, and JNK phosphorylation. (**a**) The reduction effect of lobohedleolide on the binding of C/EBPβ to the endogenous COX-2 promoter. (**b**) Lobohedleolide did not significantly reduce the binding of AP-1 to the endogenous COX-2 promoter. Ava5 cells were treated with lobohedleolide at 20 and 40 μM for 3 days. Cells were fixed with formaldehyde. AP-1- or C/EBPβ-bound DNA was isolated by chromatin immunoprecipitation with anti-AP-1 or anti-C/EBPβ antibodies, respectively. The AP-1- or C/EBP-bound COX-2 promoter DNA was analyzed by PCR. (**c**) The suppression effect of C/EBP expression and c-Jun phosphorylation by lobohedleolide in Ava5 cells. Ava5 cells were treated with lobohedleolide at the indicated concentrations for 3 days, and the total cell lysate was subjected to Western blotting with relevant antibodies. (**d**) The reduction effect of lobohedleolide on JNK phosphorylation in Ava5 cells. (**e**,**f**) Lobohedleolide did not reduce the phosphorylation level of NF-κB, ERK, and p38. Ava5 cells were treated with lobohedleolide at 40 μM. Total cell lysates were extracted at the indicated time points and subjected to Western blotting with relevant antibodies. The relative blot intensities were quantified by densitometric scanning. GAPDH served as an equal loading control in Western blotting. with relevant antibodies. The relative blot intensities were quantified by densitometric scanning. GAPDH served as an equal loading control in Western blotting.

To further examined whether lobohedleolide could reduce the binding of AP-1 and C/EBPβ to the endogenous COX-2 promoter, we performed Chomatin Immunoprecipitation assay. The result showed that lobohedleolide decreased the C/EBP binding to COX-2 promoter (Fig. 5a), but lobohedleolide did not reduce the binding of AP-1 (Fig. 5b). A previous study has shown that the activated c-Jun could control the expression of C/EBP^23^. Therefore, we further examined the levels of C/EBPβ protein and phosphorylated c-Jun in lobohedleolide-treated Ava5 cells, and the results of Western blotting showed that lobohedleolide dose-dependently reduced the C/EBPβ protein level and the phosphorylation level of c-Jun (Fig. 5c). The activated JNK is the major upstream activator of c-Jun^24^. To investigate whether the phosphorylation of JNK is involved in the inhibitory effect of lobohedleolide on COX-2 transcription, Ava5 cells were treated with lobohedleolide at 40 μM for the indicated duration ranging from 0.5 to 6 h. The results of Western blotting showed that lobohedleolide significantly reduced the phosphorylation level of JNK in a time-dependent manner (Fig. 5d). In addition to the JNK/c-Jun-C/EBPβ signaling pathway, COX-2 expression is also controlled by other transcriptional factors, including NF-κB and MAPK^25^. We found that lobohedleolide had no significant impact on the phosphorylation level of NF-κB, ERK and p38 induced by HCV (Fig. 5e,f), suggesting that the JNK/c-Jun-C/EBPβ signaling pathway is the most possible mechanism of lobohedleolide-mediated inhibition of COX-2 expression.

### Combination treatment of lobohedleolide with clinically used anti-HCV agents synergistically reduces HCV replication

To examine the potential of lobohedleolide as a supplementary therapeutic agent, we investigated the antiviral effect using combination treatments of lobohedleolide with clinically used HCV drugs, including IFN-α, the NS3/4 A protease inhibitor telaprevir, the NS5B polymerase inhibitor sofosbuvir, and the NS5A inhibitor daclatasvir. The antiviral effects of the combination treatments were compared with the inhibitory effects of single treatments of the corresponding inhibitor in Ava5 cells, resulting in three CI values (ED_50_, ED_75_, and ED_90_), through calculations as described in the Methods section. As shown in Table 1, the CI values are <1 for all ED_50_, ED_75_, and ED_90_ values, ranging from 0.12 to 0.59, showing that lobohedleolide exhibited a synergistic effect with various inhibitors on HCV replication. There was no apparent cytotoxicity in all combination treatments (data not shown).

**Table 1:** The synergistic reduction effect of lobohedleolide with IFN-α, telaprevir, or sofosbuvir on HCV replication.

Combination compound	Mean CI ± SD at			
	ED50^†^	ED75^‡^	ED90^§^	Influence
IFN-α	0.59 ± 0.06	0.38 ± 0.08	0.25 ± 0.04	Synergistic
telaprevir	0.41 ± 0.03	0.19 ± 0.02	0.12 ± 0.01	Synergistic
sofosbuvir	0.45 ± 0.03	0.31 ± 0.06	0.21 ± 0.06	Synergistic
daclatasvir	0.53 ± 0.04	0.34 ± 0.02	0.26 ± 0.05	Synergistic

Standard deviations indicate the mean ± SD of three independent experiments. ^†^50% effective dose.

^‡^75% effective dose.

^§^90% effective dose.

## Discussion

Several studies have indicated that the inhibition of COX-2 by selective inhibitors or naturally available compounds could possibly be used as a strategy for inflammation treatment, cancer prevention, and suppression of virus replication^26–28^. In the present study, we demonstrated that lobohedleolide significantly reduced the HCV-induced COX-2 transcription, protein synthesis, and its metabolite PGE_2_ production, resulting in the suppression of HCV replication (Figs 1 and 2). These observations indicate that lobohedleolide could possibly be used for the treatment of HCV-related liver diseases with chronic inflammation in addition to a potential agent to conquer HCV infection.

Resistance to DAA agents is an incoming threat in treating chronic HCV infection with long-term treatment due to high mutation frequencies of RNA viruses. Therefore, host-targeting antiviral agents can be considered as an alternative strategy for overcoming the viral resistance due to the extremely low mutation rate of host genome in eukaryotic cells and the increasing drug susceptibility in all genotypes and serotypes^29^. In addition, cocktail therapy has become a promising strategy to increase SVR and reduce drug resistance in HCV-infected patients^30,31^. In the present study, we suggest lobohedleolide as a suitable anti-HCV agent based on its potential to target host COX-2 expression. Furthermore, lobohedleolide exhibited a synergistic anti-HCV effect with either IFN-α or other FDA-approved DAA agents (Table 1). Accordingly, lobohedleolide might be considered as a dietary supplement for patients infected with HCV. Further investigation of the anti-HCV effect of lobohedleolide using a suitable animal model is required to analyze the potential use in HCV-infected patients.

An earlier study had demonstrated that lobohedleolide exhibited a reducing effect on LPS-induced iNOS and COX-2 expression in RAW264.7 macrophage cells^20^, but the precise anti-inflammatory mechanism of lobohedleolide was not clearly investigated. In this study, we clearly demonstrated that lobohedleolide reduced the HCV-induced COX-2 transcription level by mediating the C/EBP regulatory element (Figs 3 and 4). The C/EBP family is a homodimeric DNA-binding basic-leucine zipper (bZIP) transcription factor and consists of six proteins divided into two subgroups, namely, C/EBP-α, -β, and -δ and C/EBP-γ, -ε, and -ζ.^32^. C/EBP-β and -δ are considered as the major mediators for regulating COX-2 expression^33,34^. The effect of lobohedleolide on C/EBP isoform(s) involved in COX-2 expression in Ava5 cells remains to be investigated. In addition to C/EBP as an up-regulator for COX-2 expression during HCV infection, HCV-induced production of C/EBP mRNA causes an increase in the expression of inflammatory cytokines and chemokines, such as IL-1α, TNF-α, and CXCL1^35^, which contributes to chronic fibrogenesis and hepatocarcinogenesis^36^. Clinically, a high expression level of IL-1α, TNF-α, and IL-2 was observed in HCV-infected patients^37^. In addition, the expression of a wide range of genes and microRNAs, such as miR-181c and miR-122, are also regulated by C/EBP, which participate in cell growth and proliferation in HCV-related liver diseases or HCC^38,39^. Further investigation of the inhibitory effect of lobohedleolide on C/EBP-regulated inflammation in different chronic injuries could be performed, which would provide another potential therapeutic possibility of using lobohedleolide against HCV-related diseases. In contrast to the upregulator of COX-2 expression, the WT p53 has been considered as a suppressor of COX-2 transcription as it competes with the TATA-binding protein^40^. Previous studies have shown that p53 expression is downregulated by the far upstream element (FUSE)-binding protein, leading to persistent HCV replication in most HCC tumors^41^. However, the relationship between p53 and COX-2 during HCV replication is not yet clearly verified. It will be useful to investigate the effect of lobohedleolide on p53-depedent inhibition on COX-2 expression during HCV infection.

**Figure 6:**
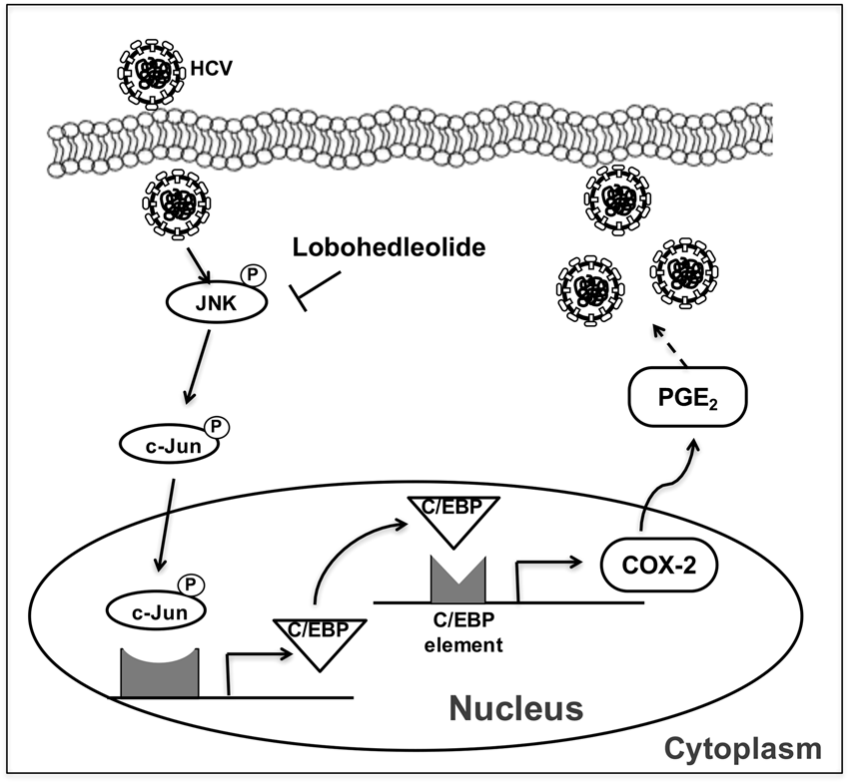
The proposed model of lobohedleolide activity against HCV replication. Lobohedleolide reduced the HCV-induced COX-2 expression by downregulating the phosphorylation of JNK resulting in attenuation of the phosphorylation of c-Jun and the expression of C/EBP. Subsequently, the suppressed level of COX-2 and PGE_2_ by lobohedleolide lad to the inhibition of HCV replication.

In conclusion, our study results have revealed that lobohedleolide suppressed HCV replication by suppressing JNK phosphorylation, leading to the downregulation of c-Jun phosphorylation and C/EBP expression. Reduced COX-2 expression by lobohedleolide contributed to the suppression of PGE_2_, finally causing a reduction on HCV RNA replication (Fig. 6). The synergistic anti-HCV effect of lobohedleolide with IFN, telaprevir, or sofosbuvir showed that lobohedleolide may serve as a therapeutic supplementary agent for increasing the treatment efficacy or decreasing the possibility of drug resistance in HCV-infected patients.

## Materials and Methods

### Cell culture and reagents

Ava5, an engineered HCV subgenomic replicon cell line, Huh-7 and Huh7.5 cells were cultured in Dulbecco’s modified Eagle’s medium (DMEM) containing 10% heat-inactivated fetal bovine serum, 1% antibiotic–antimycotic, and 1% nonessential amino acids at 37 °C with a 5% CO_2_ supplement. Ava5 cells have to be maintained in DMEM with 1 mg/ml G418 to maintain the stable expression of the replicon. Interferon alpha-2a (Roferon^®^-A) was purchase from Roche Ltd (Basel, Switzerland). Telaprevir was purchased from Legend Stat International Co., Ltd (Omdurman, Sudan). Daclatasvir and sofosbuvir was purchased from Shanghai Haoyuan Chemexpress Co., Ltd (Shanghai, China). Each experiment was incubated consistently in 0.1% dimethyl sulfoxide (DMSO). Telaprevir or sofosbuvir was stored as 10 mM in 100% DMSO.

### Preparation of lobohedleolide

Lobohedleolide was isolated from the Formosan soft coral *L*. *crassum* following a method that has been described previously^20^.

### Western blotting

Ava5 cells were seeded into a 24-well plate at a density of 5 × 10^4^ cells/well and into a 6-well plate at a density of 5 × 10^5^ cells/well. After 12–16 h of incubation, cells were treated with the reagents for appropriate duration at the indicated concentrations. Then, the cells were washed with ice-cold PBS and lysed using RIPA lysis buffer. The insoluble protein was removed by centrifugation at 12,000 rpm for 30 min at 4 °C, and the protein concentration of the soluble lysate was measured by Bio-Rad protein assay kit (Hercules, CA, USA). Immunoblotting analysis was performed as previously described. Briefly, an equal amount of protein was loaded onto 10% SDS-polyacrylamide gel electrophoresis and transferred to PVDF membranes. The levels of the protein of interest were measured using specific antibodies against HCV NS5B (1:5000; Abcam Cambridge, MA, USA), glyceraldehyde-3-phosphate dehydrogenase (GPADH), COX-2 (1:1000; Cayman, ML, USA), Myc (1:1000; GeneTex, CA, USA), C/EBP, p-c-Jun, p-p38, t-p38, p-JNK, t-JNK, p-ERK, and t-ERK (1:1000; Cell Signaling Technology, Danvers, MA, USA). The blotting signal was developed using ECL detection kit (PerkinElmer, CT, USA) and was counted by the software Quantity One (Bio-Rad, CA, USA).

### Quantitative real-time PCR (qRT-PCR) assay

The cells were incubated with lobohedleolide for appropriate duration at the indicated concentrations. Total cellular RNA was extracted using a Total RNA Miniprep Purification Kit (GMbiolab, Kaohsiung, Taiwan) according to the manufacturer’s instructions. After being transcribed to cDNA by M-MLV reverse transcriptase (Promega, Madison, WI, USA) with HCV 3ʹ UTR (5ʹ-acttgatctgcagagaggcc-3ʹ) or oligo dT primer, the level of cDNA was determined through qRT-PCR with specific primers as previously described. The CT value of each sample was determined using the ABI Step One Real-Time PCR System, which was normalized to endogenous cellular *gapdh* gene. The PCR primers were as follows: GAPDH, 5ʹ-gtcttcaccaccatggagaa-3ʹ (forward), and 5ʹ-atggcatggactgtggtcat-3ʹ (reverse); NS5B, 5ʹ-ggaaaccaagctgcccatca-3ʹ (forward), and 5ʹ-cctccacggatagaagttta-3ʹ (reverse).

### Cytotoxicity assay

Cell cytotoxicity was determined using colorimetric 3-(4,5-dimethylthiazol-2-yl)-5-(3-carboxymethoxyphenyl)-2-(4-sulfophenyl)-2H-tetrazolium (MTS) assay (Promega, Madison, WI, USA) according to the manufacturer’s instructions. Briefly, 5 × 10^3^ Ava5 cells/well were seeded into a 96 well-plate and treated with lobohedleolide at the indicated concentrations for 3 days. The cells were then treated with 20 μl MTS solution and 80 μl PBS per well for 2–4 h at 37 °C, and the relative cytotoxicity was quantified by measuring the absorbance at 490 nm using a 550 BioRad plate-reader (Bio-Rad, Hertfordshire, UK).

### HCV particle preparation and infection assay

Full-length and linearized JFH-1 RNA was transfected into Huh7.5 cells for producing infectious HCV genomic type 2a JFH-1 virus particles^42^. After 3 days, the supernatant was collected and filtered through a 0.45-μm filter and stored at −70 °C until use. The infectivity rate of HCV JFH-1 was determined through serial diluting and infecting the Huh7.5 cells for 3 days. Immunostaining was performed with anti-core antibodies. Huh7.5 cells were infected with HCV JFH-1 particles at a multiplicity of infection of 0.1 for 6 h, and the infected cells were washed with PBS and replaced with fresh medium. Lobohedleolide was applied at various concentrations for an additional 3 days. Total RNA was extracted and subjected to qRT-PCR as described above.

### Transfection and luciferase activity assay

The Ava5 cells were seeded into a 24-well plate at a density of 5 × 10^4^ cells/well and incubated for 16 h. Then, 0.2 μg of pCMV-Rellina-Luc and 1 μg of a series of COX-2 promoter reporter plasmids, including pCOX-2-Luc, pCOX-2-(ΔNF-κB)-Luc, and pCOX-2-(ΔNF-κB/C/EBP)-Luc were cotransfected into Ava5 cells by T-pro reagent (Ji-Feng Biotechnology CO., Ltd., Taipei, Taiwan) for 6 h following the manufacturer’s instructions. To investigate the critical transcription factor involved in the inhibition of COX-2 expression by lobohedleolide, Ava5 cells were cotransfected with 0.2 μg of pCMV-Rellina-Luc and pAP-1-Luc, pNF-κB-Luc, or pC/EBP-Luc for 6 h. pCMV-Rellina-Luc served as the internal control for evaluating the transfection efficiency. The cells were exchanged with fresh medium containing lobohedleolide at the indicated concentrations. After 3 days of incubation, the cell extracts were subjected to luciferase activity assay using Dual-Glo Luciferase Assay System (Promega, Madison, WI, USA) in accordance with the manufacturer’s instructions. pAP-1-Luc, pNF-κB-Luc and pC/EBP-Luc were purchased from Stratagene. Under exogenous gene expression condition, either vehicle vector pcDNA4/myc-His-A (Life Technologies, Carlsbad, CA, USA) or pcDNA4-COX-2-myc-His was transfected into Ava5 cells at various concentration (0.5, 1.0 and 1.5 μg) for 6 h. The cells were exchanged with fresh medium containing lobohedleolide at 40 μM for 3 days. The cell lysate and cellular RNA were analyzed by Western blotting and qRT-PCR as previously described, respectively.

### Chromatin Immunoprecipitation assay

The ability of C/EBPβ to bind to COX-2 promoter upon lobohedleolide treatment was examined using Magna ChIPTM A/G kit according to the manufacturer’s protocol (Merck, Darmstadt, Germany). Briefly, Ava5 cells were treated with lobohedleolide for 3 days. Cells were then cross-linked with 1% formadehyde for 10 minutes. After washing with PBS, cells were lysed with Cell Lysis Buffer containing protease inhibitor cocktail. The chromatin was sheared by sonication. 100 ng of DNA were used as input. The remaining chromatin fraction were immunoprecipitated with anti-C/EBPβ (4 μg; GeneTex, CA, USA) or anti-AP-1 antibodies (10 μg; Sigma, MO, USA) overnight at 4 °C. The C/EBPβ-bound COX-2 promoter DNA was analyzed by PCR with specific primers: 5ʹ-cggagggtagttccatgaaa-3ʹ (forward), and 5ʹ-caggcttttacccacgcaaa-3ʹ (reverse). PCR was perfomed at 94 °C for 30 s, 59 °C for 30 s, and 72 °C for 30 s, for 35 cycles.

### Analysis of the drug synergism

Ava5 cells were seeded into a 24-well plate at a density of 5 × 10^4^ cells/well and treated with serially diluted lobohedleolide at 2.5, 5, 10 and 20 μM in combination with diluted IFN-α (7.5, 15, 30, and 60 U/mL), the HCV protease inhibitor telaprevir (0.075, 0.15, 0.3, and 0.6 μM), or the RNA-dependent RNA polymerase nucleoside inhibitor sofosbuvir (10, 20, 40, and 80 nM). Each of the combination treatment was performed by adding lobohedleolide horizontally with various HCV inhibitors vertically in a checkerboard cross in the 24-well plate. After 3 days of treatment, total cellular RNA was extracted, and the RNA levels were quantified by qRT-PCR with specific primers as described above. The combination index (CI) values of each combination achieving 50%, 75%, or 95% reduction in the HCV RNA level were calculated using the CalcuSyn2TM computer program (Biosoft, Cambridge, UK), which was based on the Chou and Talalay analysis method^43,44^. Primarily, CI values of 1, <1, and >1 indicate additive, synergistic, and antagonistic effects, respectively.

### Statistical analysis

All data were obtained from at least three independent experiments and are presented as mean ± SD. Statistical significance was determined using Student’s *t* test for differences between two data groups (lobohedleolide-treated and -untreated cells). *P < 0.05 or **P < 0.01 was considered to be statistically significant.

## Electronic supplementary material

Supplementary Information
