# Supplementary material for: Lobohedleolide suppresses hepatitis C virus replication via JNK/c-Jun-C/EBP-mediated down-regulation of cyclooxygenase-2 expression
Source: Sci Rep. 2018 Jun 6;8:8676. doi: 10.1038/s41598-018-26999-w (PMC5989199; doi:10.1038/s41598-018-26999-w)

Lobohedleolide suppresses hepatitis C virus replication via JNK/c-Jun-C/EBP-mediated down-regulation of cyclooxygenase-2 expression

Chun-Kuang Lin^1, 2^, Chin-Kai Tseng^3, 4^, Chih-Chuang Liaw^1, 5^, Chiung-Yao Huang^5^, Chih-Ku Wei^6^, Jyh-Horng Sheu^1, 5, 7^*, Jin-Ching Lee^6, 8, 9, 10^*

^1^Doctoral Degree Program in Marine Biotechnology, College of Marine Sciences, National Sun Yat-Sen University, Kaohsiung, Taiwan

^2^Doctoral Degree Program in Marine Biotechnology, Academia Sinica, Taipei, Taiwan

^3^Institute of Basic Medical Sciences, College of Medicine, National Cheng Kung University, Tainan, Taiwan

^4^Center of Infectious Disease and Signaling Research, College of Medicine, National Cheng Kung University, Tainan, Taiwan

^5^Department of Marine Biotechnology and Resources, College of Marine Sciences, National Sun Yat-Sen University, Kaohsiung, Taiwan

^6^Department of Biotechnology, College of Life Science, Kaohsiung Medical University, Kaohsiung, Taiwan

^7^Department of Medical Research, China Medical University Hospital, China Medical University, Taichung, Taiwan

^8^Graduate Institute of Medicine, College of Medicine, Kaohsiung Medical University, Kaohsiung, Taiwan.

^9^Research Center for Natural Products and Drug Development, Kaohsiung Medical University, Kaohsiung, Taiwan.

^10^Department of Medical Research, Kaohsiung Medical University Hospital, Kaohsiung, Taiwan

**Running title:** Lobohedleolide suppresses hepatitis C virus replication

***Corresponding authors:** Jin-Ching Lee and Jyh-Horng Sheu

**Mail address:** Department of Biotechnology, Kaohsiung Medical University, 100, Shih-Chuan 1^st^ Road, San Ming District, 807 Kaohsiung City, Taiwan.

**Phone:** 886-7-312-1101 ext 2369 **Fax:** 886-7-312-5339

**E-mail:** [jclee@kmu.edu.tw](mailto:jclee@kmu.edu.tw) and [sheu@mail.nsysu.edu.tw](mailto:sheu@mail.nsysu.edu.tw)

**Supplementary information**

**The original full length immunoblots related to Figure 1B and 1D.**


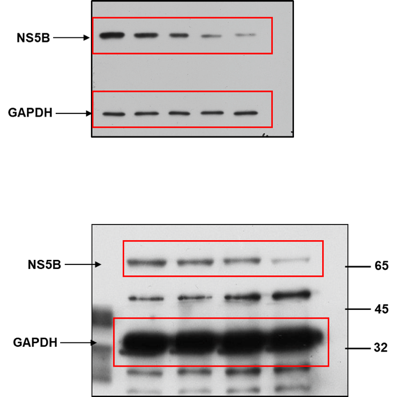


**The original full length immunoblots related to Figure 2A, 2B and 2F.**

**
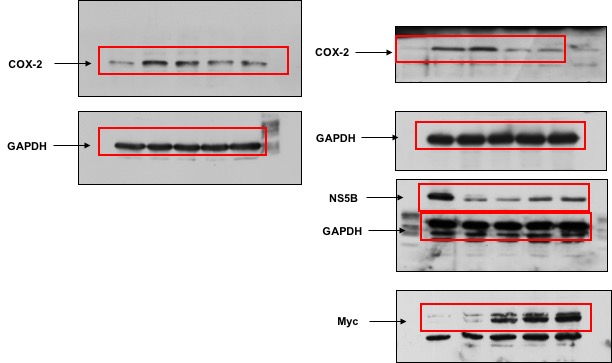
**

**The original full length immunoblots related to Figure 5**


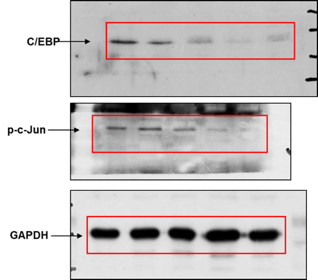


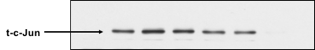


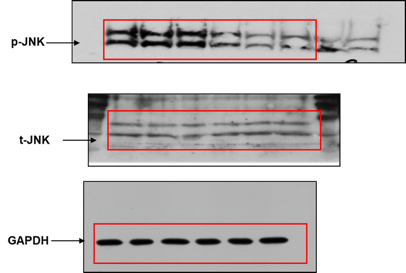


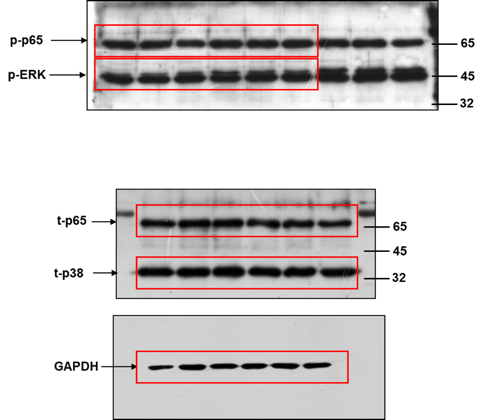


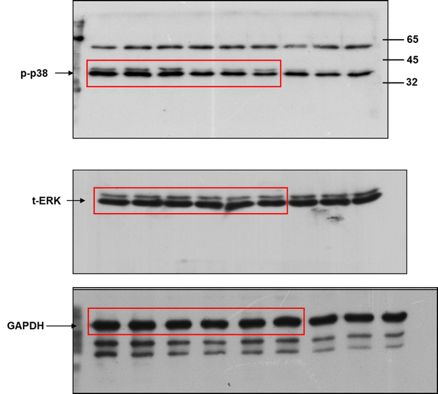

Supplement: Supplementary file 1 — Supplementary Information [file 41598_2018_26999_MOESM1_ESM.docx]
